# MYC is downregulated by a mitochondrial checkpoint mechanism

**DOI:** 10.18632/oncotarget.21653

**Published:** 2017-10-06

**Authors:** Xiaonan Zhang, Arjan Mofers, Per Hydbring, Maria Hägg Olofsson, Jing Guo, Stig Linder, Padraig D'Arcy

**Affiliations:** ^1^ Cancer Center Karolinska, Department of Oncology and Pathology, Karolinska Institute, SE-171 76 Stockholm, Sweden; ^2^ Department of Medical and Health Sciences, Linköping University, SE-581 83 Linköping, Sweden; ^3^ Department of Medical Biochemistry and Biophysics (MBB), Karolinska Institute, SE-171 77 Stockholm, Sweden; ^4^ Cardiovascular and Metabolic Disorders Program, Duke-NUS (National University of Singapore) Medical School, 16957 Singapore, Singapore

**Keywords:** mitochondria, MYC, CRD-BP, let-7/miR-34a, cancer

## Abstract

The MYC proto-oncogene serves as a rheostat coupling mitogenic signaling with the activation of genes regulating growth, metabolism and mitochondrial biogenesis. Here we describe a novel link between mitochondria and MYC levels. Perturbation of mitochondrial function using a number of conventional and novel inhibitors resulted in the decreased expression of MYC mRNA. This decrease in MYC mRNA occurred concomitantly with an increase in the levels of tumor-suppressive miRNAs such as members of the *let-7* family and *miR-34a-5p*. Knockdown of *let-7* family or *miR-34a-5p* could partially restore MYC levels following mitochondria damage. We also identified *let-7*-dependent downregulation of the MYC mRNA chaperone, CRD-BP (coding region determinant-binding protein) as an additional control following mitochondria damage. Our data demonstrates the existence of a homeostasis mechanism whereby mitochondrial function controls MYC expression.

## INTRODUCTION

The cell cycle is a finely orchestrated event dependent on the sequential activation of genes regulating cell growth and metabolism. The proto-oncogene c-*MYC* (referred to as *MYC*) represents a master regulator via integration of external mitogenic signaling with the activation of genes required for cell cycle progression [1]. Although inherently unstable, growth factor signaling leads to increased MYC levels and its association with MAX forming a transcriptional heterodimer complex that binds to conserved E-box elements (consensus CACGTG) in the promoters of target genes leading to gene activation [1]. A large number of E-box containing genes have been identified underlying the important role of MYC as a master regulator of gene expression [2]. Genes encoding proteins involved in cell growth, metabolism, ribosome biogenesis, protein synthesis and mitochondrial function have all be defined as potential MYC targets. In spite of such a broad range of potential targets, the MYC protein is surprisingly a relatively weak transcriptional activator and is presumed to function as a general enhancer of transcription [3, 4].

Considering multiple signaling pathways converge on MYC it is not surprising that overexpression is frequently observed in multiple tumor types where elevated MYC levels are associated with malignant transformation and tumor proliferation [5]. Furthermore depletion of MYC using shRNA leads to proliferative arrest in multiple tumor cell lines [6, 7]. Not surprisingly, inhibition of MYC has been suggested as an anti-cancer therapy. While the role of MYC in cell cycle progression has been well established, the role of MYC in regulating metabolism is less well understood. Several groups have demonstrated an important role for MYC in controlling cellular energy demand via mitochondrial biogenesis [8–11]. MYC regulates mitochondrial mass and biogenesis by enhancing the expression of genes regulating mitochondrial fusion and fission cycles [9].

Changes in mitochondria function leads to signaling events that are transduced to the rest of the cell, by a process commonly referred to as “mitochondrial retrograde signaling” [12]. Signaling cascades induced by changes in mitochondrial bioenergetics capacity are important for the maintenance of cellular homeostasis. In yeast cells mitochondrial dysfunction induces altered gene expression to compensate for reduced metabolic activity and decreased ATP production [12]. In cultured mammalian cells, several different mitochondrial retrograde signaling mechanisms have been described, including the activation of Ca^2+^-signaling, the release of metabolites, ROS production and activation of the mTOR pathway [12–15]. The type of mitochondrial dysfunction is known to influence the pattern of nuclear gene expression and, presumably, the mechanism of retrograde signaling [16].

MicroRNAs (miRNAs) are small noncoding RNAs that dampen gene expression by binding to complementary nucleotide sequences in mRNA and promoting degradation and/or repression of translation initiation, thus leading to the altered regulation of a wide variety of biological processes [17–19]. The *let-7* family is a highly conserved group of *miRNA* genes that were among the first to be described in mammalian cells [20]. Several studies have shown that *let-7* functions as a tumor suppressor miRNA by inhibiting the expression of growth promoting proto-oncogenes, such as *RAS* and *MYC* [21, 22] or by the destabilization of mRNA chaperones such as IMP-1/CRD-BP (coding region determinant-binding protein) [23]. *miR-34a-5p* shares a similar function with the *let-7* family and acts as a potent tumor suppressor by altering the stability of growth promoting oncoproteins [24, 25]. In addition both *let-7* and *miR-34a* share overlapping target sites in the 3’UTR of *MYC* mRNA, thus act as negative regulators of MYC expression [26].

The requirement of MYC for tumor cell proliferation has led to an interest in developing compounds that inhibit MYC [5]. Several MYC inhibitors have been identified from phenotypic screens, including 10058-F4, atorvastatin and nitazoxanide [27–30]. 10058-F4 disrupts the interaction between MYC/MAX blocking cellular proliferation [27, 28], atorvastatin decreases MYC phosphorylation and activity [29] and nitazoxanide reduces MYC protein expression and shows *in vivo* antineoplastic activity [30].

We recently identified VLX600 from a phenotypic screen of compounds that induce apoptosis of non-proliferating cells in 3-D multicellular spheroids [31]. The anti-proliferative effects of VLX600 were primarily due to alterations in mitochondrial activity caused by the reduced expression of cytochrome c oxidase subunit 1 (COX-I) and inhibition of mitochondrial oxidative phosphorylation (OXPHOS) [31]. In this study we identify an unexpected effect of VLX600 exposure, namely the down regulation of MYC expression. Interestingly this phenomenon on MYC expression was also observed following exposure to other mitochondrial inhibitors, suggesting that MYC expression is controlled by mitochondrial activity. We examined the mechanism of MYC down regulation by VLX600 and found that it occurred at the level of decreased mRNA stability and up-regulation of tumor suppressive *let-7a* family and *miR-34a-5p* miRNAs. Taken together, these data identify down regulation of MYC expression as a mitochondrial retrograde signaling event.

## RESULTS

### The mitochondrial inhibitor VLX600 downregulates MYC expression

**Figure 1:**
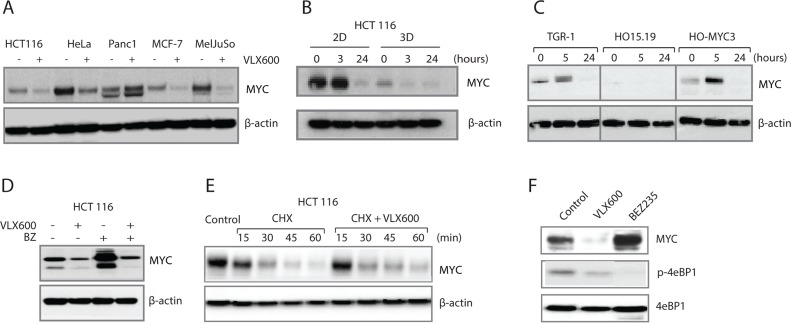
VLX600 decreases MYC protein expression **(A)** Human tumor cells were exposed to 6.5 μM VLX600 for 24 hours followed by western blot analysis for MYC and β-actin. **(B)** Monolayer and MCTS of HCT116 colon cancer cells were treated with 6.5 μM VLX600 followed by western blot analysis for MYC and β-actin. **(C)** TGR-1, HO15.19 and HO-MYC3 rat fibroblasts were treated with 6.5 μM VLX600 followed by western blot analysis for MYC and β-actin. The *MYC* gene is deleted by gene targeting in HO15.19 cells; HO-MYC3 is a derivative of HO15.19 where MYC is expressed by a retrovirus vector [32]. **(D)** HCT116 cells were treated with 6.5 μM VLX600 in the presence or absence of 100 nM bortezomib (BZ) for 24 hours followed by western blot analysis for MYC and β-actin. **(E)** HCT116 cells were exposed to 50 μg/mL cycloheximide (CHX) in the presence or absence of 6.5 μM VLX600 followed by western blot analysis for MYC and β-actin. **(F)** HCT116 cells were treated with VLX600 (6.5 μM) or BEZ235 (0.2 μM) for 24 hours and processed for western blotting using MYC, p-4eBP1 and phospho-4eBP1 antibodies.

We recently identified the compound VLX600 as a small molecule capable of inducing apoptosis in the quiescent compartment of 3D tumor multicellular tumor spheroids (MCTS) [31]. A curious observation from our initial findings was the down-regulation of cytochrome c oxidase subunit 1 (COX-I) protein levels that occurred concomitantly with a reduction in mitochondrial oxidative phosphorylation (OXPHOS) capacity following exposure to VLX600 [31]. As a potential explanation for this mechanism we turned our attention to the *MYC* proto-oncogene since previous studies have shown that MYC activates the transcription of nuclear encoded mitochondrial genes, including COX-1 [8]. Treatment of a panel of human carcinoma cell lines with VLX600 resulted in a strong reduction in MYC levels in 4/5 cell lines tested (Figure 1A). Comparison of MYC expression in 2D cultures vs. 3D MCTS showed that MYC levels were generally lower in the 3D MCTS, which is consistent with the presence of high numbers of non-proliferating quiescent cells in the MCTS core. However, in spite of this the levels of MYC in MCTS could be reduced following VLX600 exposure (Figure 1B, Supplementary Figure 1). This phenomenon was not confined to solely human cells since a similar reduction in MYC protein levels and was also observed in TGR-1 rat fibroblasts following drug exposure (Figure 1C). Interestingly, a similar pattern in reduction of MYC levels by VLX600 was observed in the rat cell line HO-myc3 (derived from HO15.19, a MYCnull cell line from TGR-1 cells) where MYC is expressed under the control of a retrovirus promoter [32], suggesting that alterations in expression were not at the level of the endogenous MYC promoter.

MYC expression is regulated at multiple levels, including transcription initiation, mRNA stability, translation and protein turnover [33]. In order to investigate if VLX600 increases the rate of MYC protein degradation we co-treated cells with the proteasome inhibitor bortezomib. While bortezomib increased the basal levels of MYC, a reduction following VLX600 exposure was still observed (Figure 1D), suggesting that changes in ubiquitin-mediated degradation were unlikely to account for the alterations in MYC expression. We also investigated if the downregulation of MYC observed following treatment with VLX600 was due to degradation by alternative pathways; however MYC expression could not be rescued by the addition of the autophagy inhibitor chloroquine (Supplementary Figure 2).

We next examined if VLX600 altered the half-life of the MYC protein by performing a cycloheximide chase, where nascent MYC translation was blocked by the addition of cycloheximide to the cell culture media. We observed no changes in the half-life of the MYC protein in the presence of VLX600 (Figure 1E), suggesting that VLX600 does not alter MYC protein stability. We previously showed that mTOR signaling is inhibited by VLX600 [31]. Since mTOR has been shown to increase the efficacy of *MYC* mRNA translation [34, 35], we examined whether the PI3K/mTOR inhibitor BEZ235 would decrease MYC expression in HCT116 cells. However, and consistent with a previous report [36], MYC expression was increased in BEZ235-treated HCT116 cells (Figure 1F), suggesting that the effect of VLX600 on MYC expression is unlikely to be mediated by mTOR inhibition.

### VLX600 decreases MYC mRNA stability and decreases CRD-BP levels

**Figure 2:**
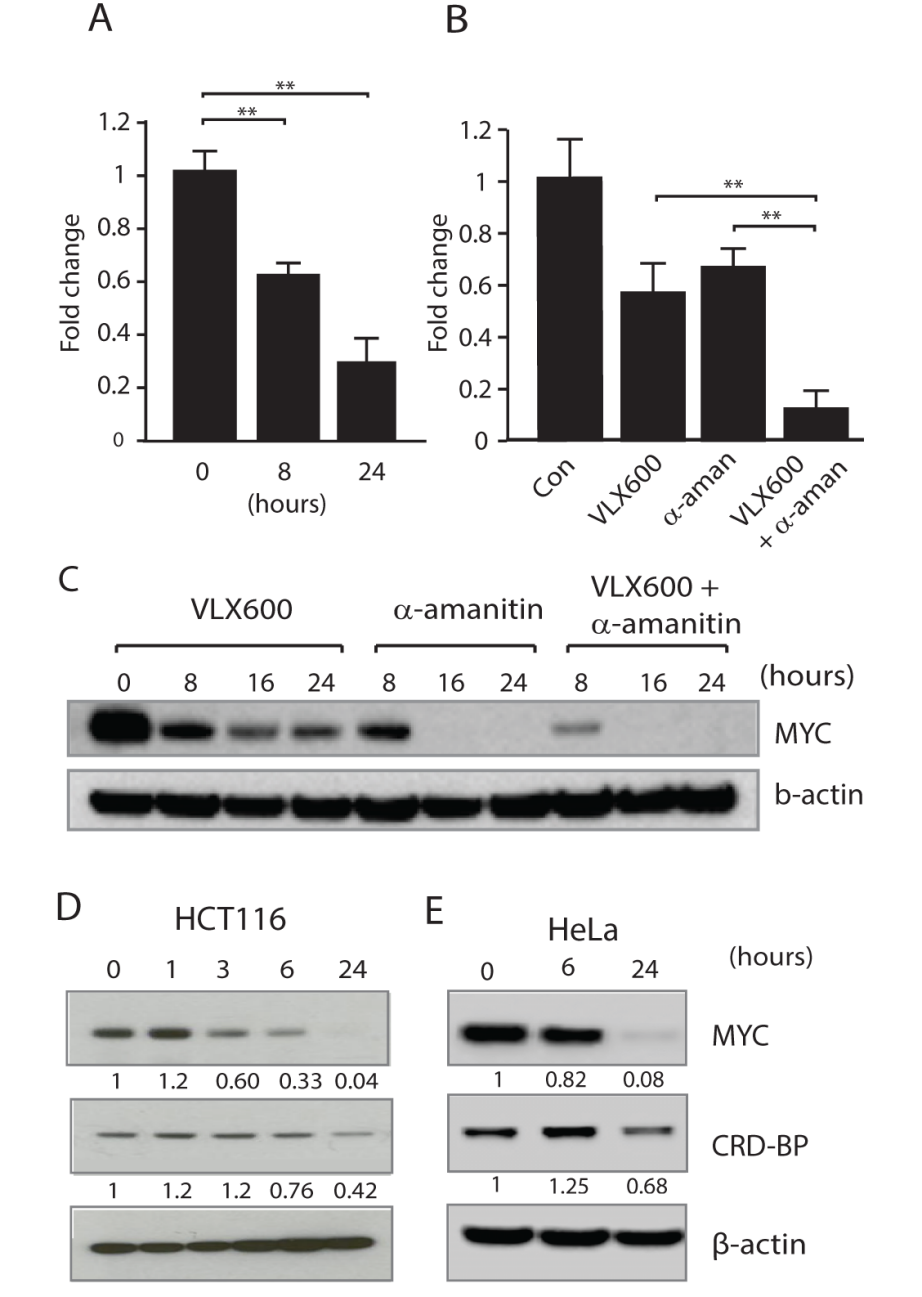
Reduced MYC expression is associated with decreased MYC mRNA stability **(A)** HCT116 cells were exposed to 6.5 μM VLX600 for 8 or 24 hours and *MYC* mRNA levels were determined using RT-PCR. Tubulin was used as an internal control and results shown as changes relative to untreated control (^**^ p < 0.01). **(B)** HCT116 cells were incubated in the presence or absence of 6.5 μM VLX600 and/or 10 μM α-amanitin for 8 hours and *MYC* mRNA levels determined by RT-PCR using tubulin as the internal control (^**^ p < 0.01). **(C)** HCT116 cells were incubated in the presence or absence of 6.5 μM VLX600 and/or 10 μM α-amanitin for the indicated times followed by western blot analysis for MYC and β-actin. **(D, E)** HCT116 or HeLa cells were treated with 6.5 μM VLX600 for the indicated times, followed by western blot analysis for MYC, CRD-BP and β-actin. Numbers below each panel: quantification relative to loading control. All experiments were repeated at least 3 times.

We next examined the effect of VLX600 on *MYC* mRNA levels. Exposure to VLX600 resulted in significant reductions of *MYC* mRNA levels (Figure 2A). *MYC* mRNA is inherently unstable and is subject to posttranscriptional regulation [37, 38]. To examine whether VLX600 decreases *MYC* mRNA stability, we utilized α-amanitin, a potent inhibitor of RNA polymerase II. While VLX600 reduced *MYC* mRNA levels ~2-fold over an 8 hour period, the combination of α-amanitin and VLX600 resulted in a > 5-fold decrease (Figure 2B), supporting the notion that VLX600 exerts its anti-MYC effect via decreased mRNA stability. This effect was also apparent on the protein level (Figure 2C).

*MYC* mRNA contains a 249-nucleotide instability element known as the coding region determinant or CRD [39, 40]. The CRD element is subject to endonuclease attack unless protected by association with the CRD-binding protein (CRD-BP). We examined whether reductions in the levels of CRD-BP following VLX600 treatment could account for the decrease in *MYC* mRNA levels. In support of this we found a decline in the levels of CRD-BP following 24 h exposure of HCT116 (~60% reduction) or HeLa (~30% reduction) cells to VLX600 (Figure 2D and 2E) suggesting that the reduction in MYC levels observed, were at least partially due to reduced CRD-BP levels.

### VLX600 alters the expression of miRNAs

**Figure 3:**
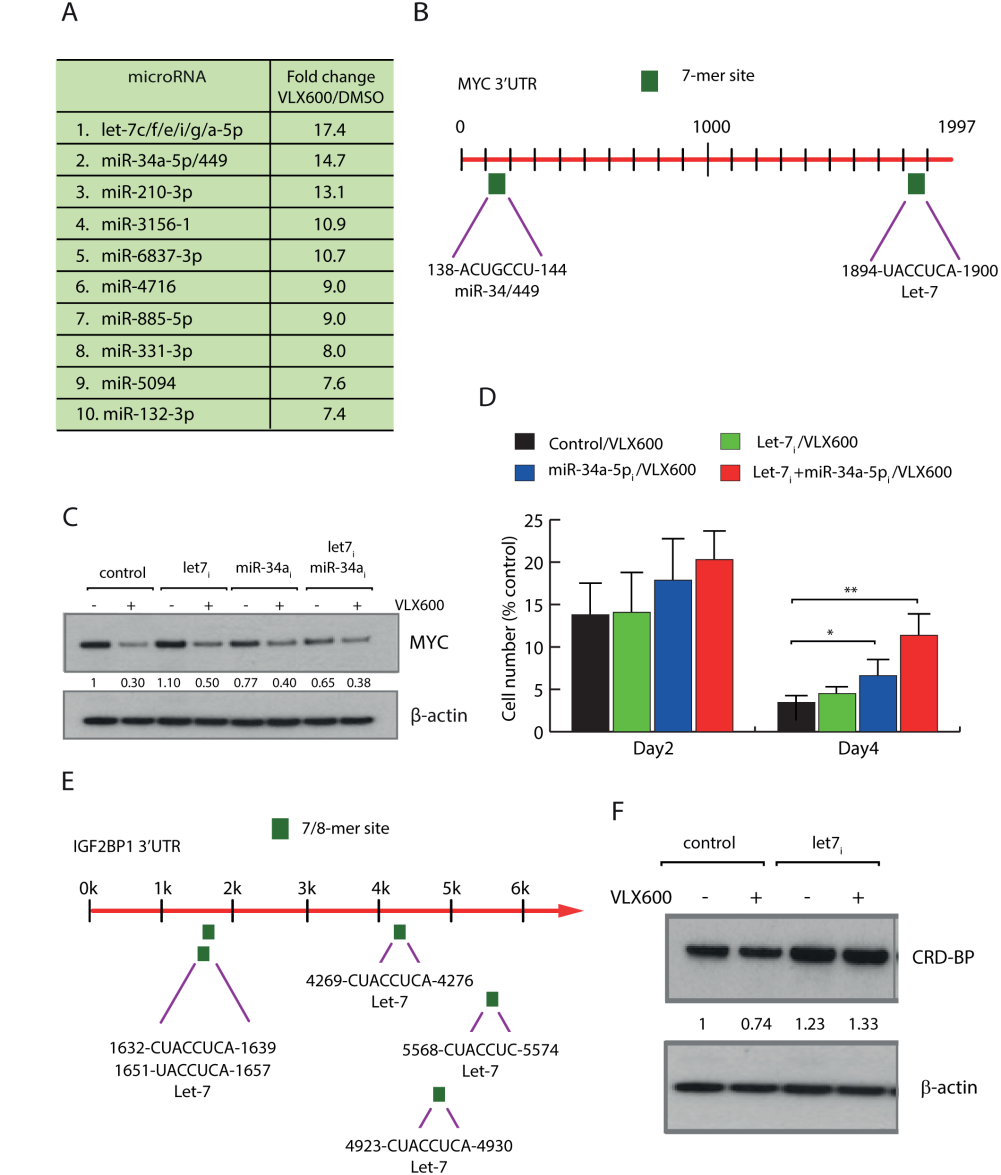
Regulation of MYC at the level miRNAs **(A)** HeLa cells were treated with 6.5 μM VLX600 for 12 hours, followed by miRNA array analysis. Shown are fold-changes in expression relative to vehicle-treated control. miRNAs with identical seed-sequences were analyzed as single entities. **(B)** Canonical miRNA binding sites of the *let-7* and *miR-34/449* families in the MYC 3’UTR are indicated. **(C)** HeLa cells were transfected with *let-7* family inhibitor or *miR-34a-5p* inhibitor for 24 hours as indicated, followed by treatment with 6.5 μM VLX600 for another 24 hours. MYC and β-actin levels were determined by western blotting; numbers indicate MYC/β-actin ratios. **(D)** HeLa cells were transfected with miRNA inhibitors and treated in the presence or absence of 6.5 μM VLX600 for 2 or 4 days. The numbers of viable cells were determined and shown as a percent of control (vehicle-treated cells). ^*^ p < 0.05; ^**^ p < 0.01. **(E)** Structure of the *IGF2BP1* 3'UTR, potential binding sites for *let-7* are indicated. *IGF2BP1* encodes the CRD-BP protein. **(F)** HeLa cells were transfected with *let-7* family inhibitor and exposed to VLX600 as indicated. CRD-BP and β-actin levels were determined by western blotting; numbers indicate CRD-BP/β-actin ratios.

The reductions of MYC protein levels were difficult to explain solely by effects mediated by CRD-BP. Because decreases in *MYC* mRNA and MYC protein were observed earlier than decreases in CRD-BP and were more pronounced (see Figure 2). We therefore postulated the existence of an alternative miRNA-dependent mechanism contributing to decreased MYC expression levels and examined the cellular miRNA pool in HeLa cells following exposure to VLX600. Increased levels of a number of miRNAs were observed following VLX600 treatment (Figure 3A). Of interest, the expression of *let-7* family miRNAs, miRNAs sharing the seed-sequence GAGGUA in the 5’ processed miRNA arm, and *miR-34a-5p* miRNAs, both of which have been described to target *MYC* [41, 42], were most strongly altered. The *MYC* transcript contains a 3’UTR of 1997 nucleotides. This 3’UTR harbors one canonical binding site for the *let-7* family and one canonical binding site for the *miR-34/449* family according to TargetScanHuman Release 7.1 (Figure 3B). We examined the effects of transfection of HeLa cells with hsa-let-7 miRNA family inhibitor or hsa-miR-34a-5p miRNA inhibitor on the response of MYC expression to VLX600. Interestingly, inhibitors of *let-7* miRNA family or *miR-34a-5p* were able to reduce the VLX600-mediated effects on MYC protein expression (Figure 3C). Co-transfection with both miRNA inhibitors resulted in a partial rescue of MYC levels. These data suggest that both *let-7* miRNA family and *miR-34a-5p* are involved in regulating MYC levels following VLX600 treatment. We also examined whether inhibition of *let-7* miRNA family and *miR-34a-5p* decreases the anti-proliferative effect of VLX600. Interestingly, the inhibitory effect of VLX600 on cell proliferation was partially, but significantly, abrogated following co-transfection of *let-7* miRNA family and *miR-34a-5p* inhibitors (Figure 3D). It has previously been shown that CRD-BP mRNA is a target of *let-7* [43]. There are indeed 5 potential binding sites for *let-7* in the CRD-BP mRNA sequence (Figure 3E; the gene name for CRD-BP is *IGF2BP1*). We found that the modest reduction (~25%) of CRD-BP expression by VLX600 in HeLa cells was abrogated by inhibition of *let-7* miRNA (Figure 3F). This result raises the possibility that *let-7* decreases MYC expression at two levels, both directly on the MYC 3' UTR and indirectly via CRD-BP. Considering the higher number of *let-7* binding sites in the CRD-BP mRNA sequence, it is possible that the major effect of *let-7* on MYC is mediated via CRD-BP.

### Inhibition of MYC expression by mitochondrial inhibitors

**Figure 4:**
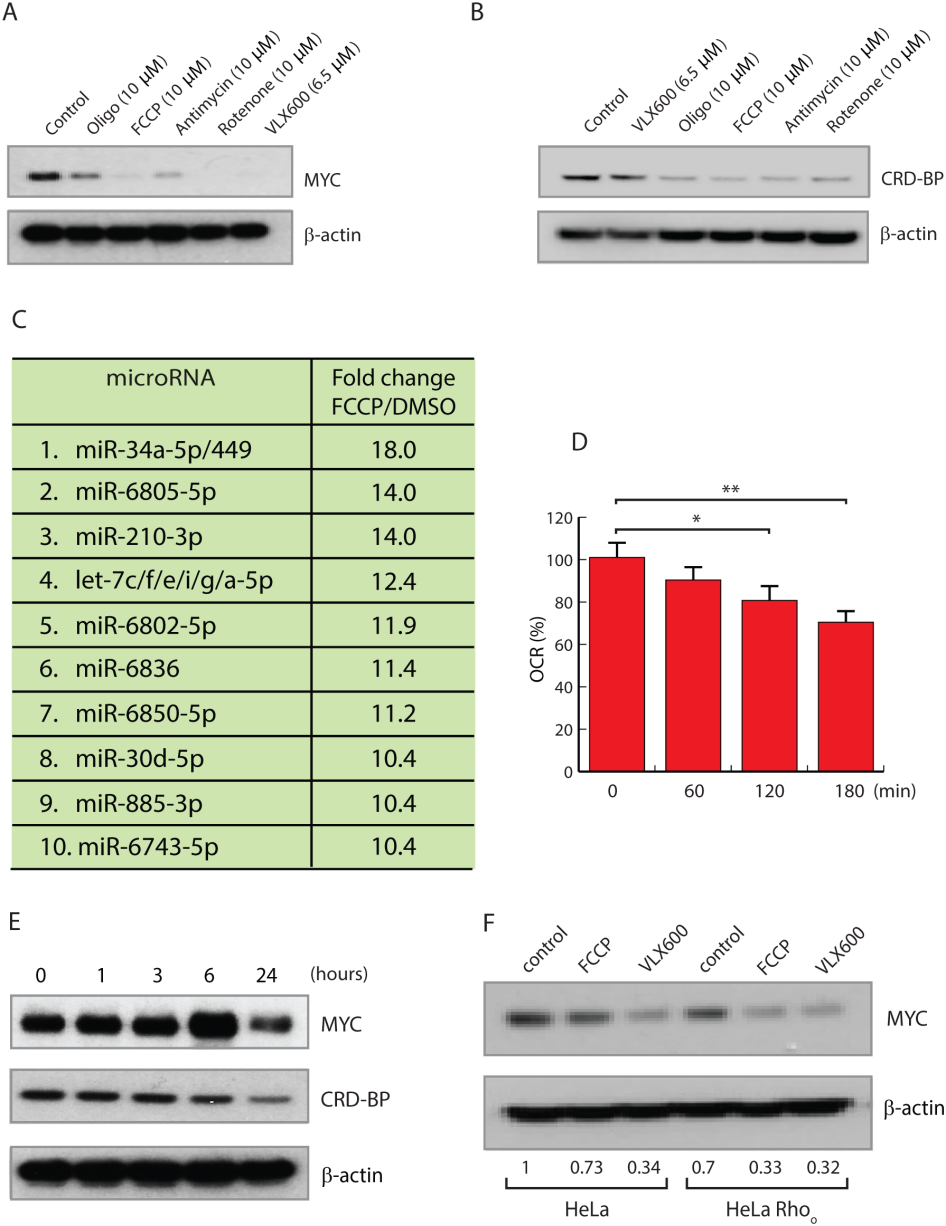
Mitochondrial inhibitors reduce MYC expression **(A, B)** HCT116 cells were exposed to 10 μM oligomycin, 10 μM FCCP, 10 μM rotenone, 10 μM antimycin or 6.5 μM VLX600 for 24 hours, followed by western blot analysis for MYC, CRD-BP and β-actin. **(C)** miRNA array analysis of HeLa cells treated with 10 μM FCCP for 12 hours. Results are normalized to treatment with vehicle and shown as fold change. miRNAs with identical seed-sequences were treated as single entities. **(D)** Oxygen consumption rate (OCR) was measured using a Seahorse XF analyzer after treatment of HCT116 cells with 5 μM salinomycin. Shown are average numbers ± SD; ^*^ p < 0.05, ^**^ p < 0.001. **(E)** HCT116 cells were treated with 5 μM salinomycin for the indicated times followed by western blot analysis for MYC and β-actin. **(F)** Expression levels of MYC in HeLa and *Rho0* cells following treatment with 10 μM FCCP or 6.5 μM VLX600 for 24 hours.

Changes in the functional state of mitochondria results in mitochondrial retrograde signaling, which is considered to be an essential mechanism for linking mitochondrial function with cellular homeostasis [12]. We hypothesized that the decreases in MYC expression observed here are due to a mitochondrial retrograde signaling mechanism triggered by altered mitochondrial function. To test this hypothesis, we examined the effects of a number of mitochondrial inhibitors on MYC levels. Decreased MYC expression was observed in cells exposed to oligomycin (an ATP synthase inhibitor), FCCP (an uncoupler), rotenone (a complex I inhibitor) and antimycin A (a complex III inhibitor) (Figure 4A). These different mitochondrial inhibitors were also found to reduce CRD-BP levels following disruption of mitochondrial OXPHOS (Figure 4B).

We examined the cellular miRNA pool in HeLa cells exposed to the uncoupler FCCP. In agreement with the result obtained using VLX600, *let-7* family and *miR-34a-5p* were some of the most strongly induced miRNAs (Figure 4C). We also examined the effect of salinomycin, an inhibitor of oxidative phosphorylation [44] that is also reported to inhibit the proliferation of cancer stem-like cells [45]. Salinomycin decreased the oxygen consumption of HeLa cells (Figure 4D) and decreased MYC and CRP-BP expression (Figure 4E). Taken together, inhibition of MYC expression by mitochondrial inhibitors appears to be a common feature and is likely to represent a general mitochondrial retrograde signaling event.

Finally we examined whether MYC inhibition would occur in *Rho0* cells. HeLa cells were grown in ethidium bromide, resulting in loss of mitochondrial DNA and subsequent mitochondria depletion (Supplementary Figure 3). Interestingly MYC expression was lower in the mitochondria depleted *Rho0* cells and could be reduced further by FCCP and VLX600 in *Rho0* cells (Figure 4F).

### The MYC/MAX inhibitor 10058-F4 decreases cellular oxygen consumption rates

**Figure 5:**
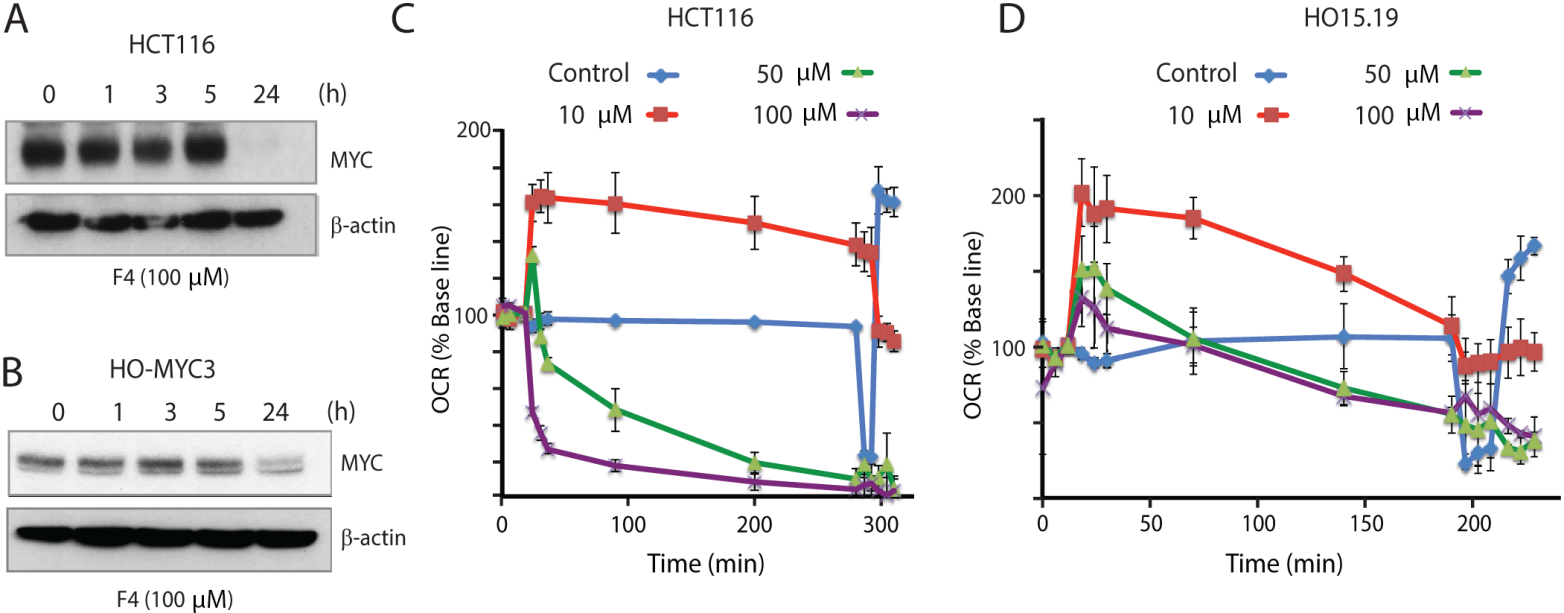
The MYC-MAX interaction inhibitor 10058-F4 alters cellular oxygen consumption **(A)** HCT116 cells were treated with 100 μM 10058-F4 for the indicated times, followed by western blot analysis for MYC and β-actin. **(B)** Rat HO-MYC3 cells were treated with 100 μM 10058-F4 for the indicated times, followed by western blot analysis for MYC and β-actin. **(C)** Oxygen consumption rates (% base line) were determined after treatment of HCT116 cells with different concentrations of 10058-F4 using a Seahorse XF analyzer. After 5 hours of treatment, oligomycin, FCCP and rotenone/antimycin were injected in sequence and changes in oxygen consumption rate (%) were recorded. Shown are means ± SD values. **(D)** Changes in oxygen consumption rates were analyzed after treatment of HO15.19 cells (*MYC*^-/-^) with different concentrations of 10058-F4 as in (C).

10058-F4 is a small molecule that has been reported to inhibit MYC/MAX interaction [28]. Based on our previous data were curious of the effect of a specific MYC inhibitor on mitochondrial function. As expected, 10058-F4 reduced MYC levels in both HCT116 and HO-myc3 cells over a 24-hour period (Figure 5A and 5B). Interestingly, it has been reported that 10058-F4-mediated down-regulation of *MYC* expression occurs via a mechanism involving the induction of *let-7a* [41].

Considering the results obtained in this study we hypothesized that 10058-F4 may decrease MYC expression as a secondary consequence of mitochondrial damage. We indeed observed rapid reductions in oxygen consumption rates (OCR) using 10058-F4 at concentrations of 50 μM and 100 μM (Figure 5C), concentrations used in other studies [41, 46, 47]. Notably, even at concentrations of 10 μM 10058-F4, OCR was decreased and displayed a pattern characteristic of mitochondrial uncoupling (rapid increase in OCR) (Figure 5C). Since the effect of 10058-F4 on OCR occurred almost immediately after injection, it was unlikely to be mediated directly by a reduction in MYC levels, since no reduction was observed in the first 5 h following exposure to 10058-F4 (Figure 5A and 5B). To further address this question, we examined the effect of 10058-F4 on the *MYC*^-/-^ cell line HO15.19. We found that 10058-F4 also decreased the OCR of these cells (Figure 5D), showing that the effect on mitochondrial OCR may be partially independent of MYC.

## DISCUSSION

In our present investigation we identify the existence of a mitochondrial retrograde signaling mechanism whereby perturbation of mitochondrial function leads to a concomitant down-regulation of MYC expression, linking MYC-regulated cell proliferation to mitochondrial integrity. We initially observed this cross talk between mitochondria and MYC via our investigations on VLX600, a novel small molecule mitochondrial inhibitor currently in phase I clinical trials for the treatment of solid tumors [31, 48] (NCT02222363, ClinicalTrials.gov). The existence of mitochondrial retrograde signaling has been extensively delineated in the budding yeast *S. cerevisiae* where perturbation of mitochondrial function induces the mitochondria-to-nucleus (Rtg) signaling pathway [12]. The Rtg pathway consists of Rtg2p, a stress responsive sensor of mitochondrial dysfunction, which subsequently activates the Rtg1p and Rtg3p transcription factors [49–51]. Oligomerization of Rtg1p and Rtg3p results in an active transcription factor complex that binds consensus motifs in target promoters resulting in the altered expression of genes regulating metabolism to counteract for the reduction in mitochondrial ATP production [49, 51]. Numerous metabolic and cellular stresses impinge on the Rtg pathway, for example, depletion of mitochondrial DNA to generate the *Rho0* phenotype activates the Rtg pathway in *S. cerevisiae* [50]. The finding that MYC levels were downregulated by VLX600 in both HCT116^wt^ and *Rho0* cells suggests the existence of an alternative mechanism of MYC regulation not strictly dependent on mitochondrial function *per se*. Since *Rho0* cells still express structurally intact, albeit non-functioning mitochondria we cannot exclude the possibility that VLX600 results in alterations in mitochondrial structure that subsequent impinge on MYC stability. Down regulation of MYC as a consequence of mitochondrial dysfunction is consistent with a “mitochondrial check-point mechanism” leading to decreased cell proliferation thus providing the cell with an opportunity to repair or generate new mitochondria before cell division occurs [52].

**Figure 6:**
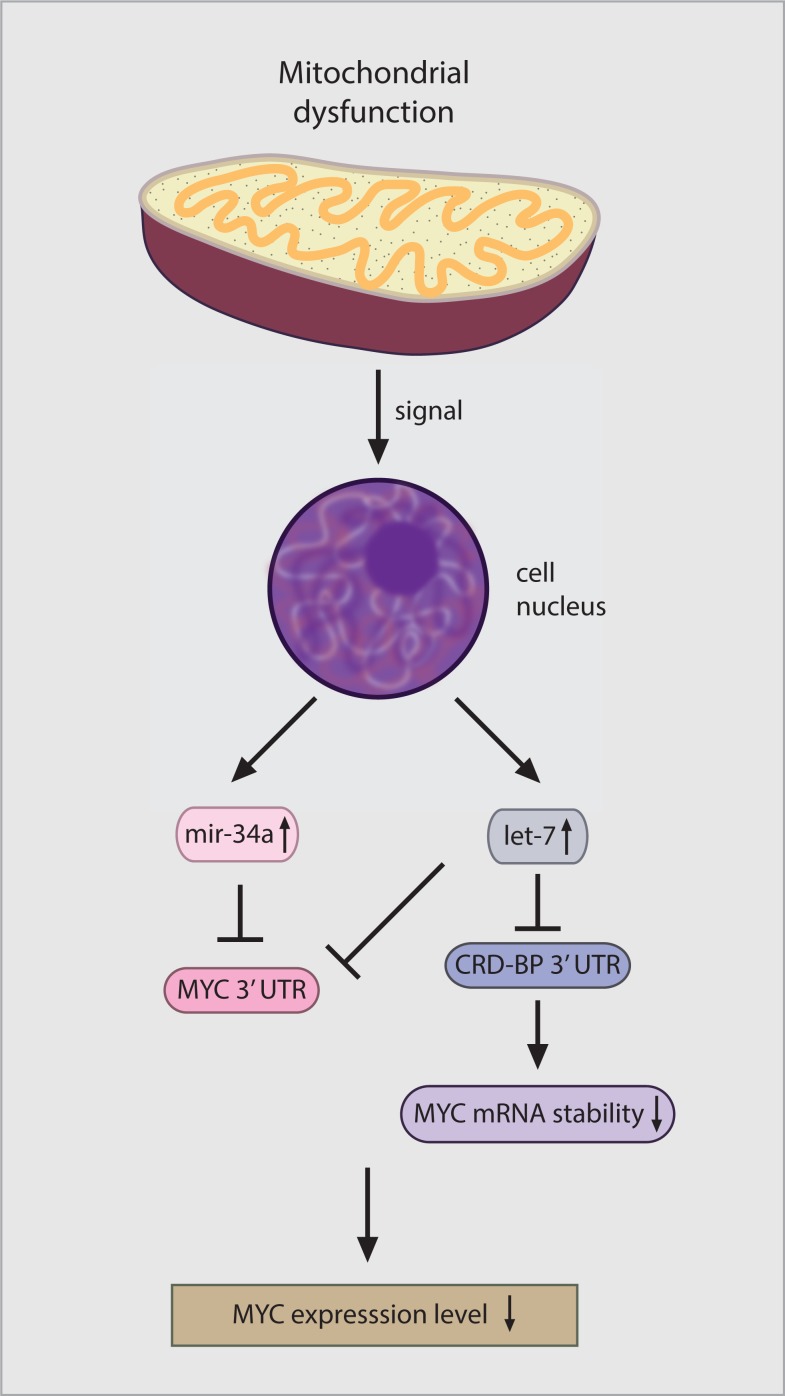
Model for how mitochondrial retrograde signaling decreases MYC expression

Our evidence suggests that the primary mode of mitochondrial regulation of MYC levels is on the level of mRNA stability. Ribosomes tend to pause at a rare arginine codon located in the C-terminal coding region of the *MYC* mRNA resulting in reduced translational efficiency and the generation of a ribosome deficient region that is susceptible to endonuclease attack unless protected by interaction with the CRD-binding protein (CRD-BP) [40]. We found decreased levels of the CRD-BP protein in cells exposed to VLX600, providing a potential mechanism for the observed reduction in MYC RNA expression. We also found that VLX600 treatment induced the expression of a number of miRNAs, including miRNAs that have been shown to regulate MYC. Considering the large number of miRNAs we focused on *let-7* and *miR-34a* since both were the highest increased miRNAs following VLX600 treatment and have been previously been identified as MYC regulators. Overexpression of miRNA *let-7a* has been reported to lead to MYC down-regulation in lymphoma cells [41]. Thus, the 10058-F4 inhibitor of MYC/MAX interaction [27] was reported to increase *let-7a* expression prior to MYC down-regulation [41]. An additional miRNA, *miR-34a* has been described as a tumor suppressor gene [53] that downregulates MYC expression [42]. We found that co-transfection with antisense reagents to *let-7a* and *miR-34a* resulted in a partial abrogation of VLX600-mediated MYC down-regulation coupled with reduced cytostatic activity of VLX600. CRD-BP is a target of *let-7* [43] and it has been reported that increased *let-7* expression reduces CRD-BP expression [54]. Induction of *let-7* may therefore be a major factor in the down regulation of MYC following mitochondrial damage, by decreasing MYC expression directly or indirectly via down regulation of CRD-BP (Figure 6). Whether other miRNAs induced by mitochondria dysfunction also play a role in MYC downregulation is an area of further study.

The small molecule 10058-F4 has been convincingly shown to bind MYC and MYCN, to inhibit the proliferation of MYC-transformed cells, to abrogate the interaction between MYCN and MAX, and to display preferential inhibitory effect on the proliferation of cells expressing MYC when compared to MYC knock out cells [27]. We found a potentially new level of 10058-F4 regulation of MYC function dependent on mitochondrial activity. Interestingly, 10058-F4 induced phenotypic changes characteristic of mitochondrial uncouplers, including a transient increase followed by decrease in OCR. Zirath *et al.* reported that 10058-F4 induces mitochondrial dysfunction and suggested this effect was caused by a down-regulation of MYC levels and altered expression of genes regulating metabolism [47]. In the current study we raise the possibility of another level of 10058-F4 inhibition of MYC, namely induction of mitochondrial dysfunction resulting in loss of MYC expression. Interestingly while c-MYC negative cells also showed reduced OCR, this effect was slower and likely represents differences in metabolism between cell lines. When all data is considered, it appears likely that the anti-proliferative effects observed are due to a combination of direct MYC inhibition, and altered mitochondrial function. Nitazoxanide is another recently described MYC inhibitor identified from a cell based screen [30]. Interestingly we have shown that nitazoxanide also induces a rapid increase in OCR similar to 10058-F4, an effect observed following mitochondrial uncoupling [44]. The exact mechanism of how miRNA expression is altered by mitochondrial inhibitors is unclear and requires further elucidation. However since many cancer cells shows MYC overexpression and/or copy number changes in the *MYC* gene, the development of agents targeting mitochondrial function may provide a therapeutic option for the treatment of cancer. In addition, our data has shown a connection between mitochondria and several tumor suppressive microRNAs including members of the *let-7* family, which could provide a possible direction for future studies on mechanisms that control the expression of *let-7* family miRNAs.

## MATERIALS AND METHODS

### Materials

All compounds were dissolved in dimethylsulphoxide (DMSO) and used at a final concentration of 0.5% DMSO; control wells received solvent only (final concentration 0.5% DMSO).

### Cell culture

HCT116 colon carcinoma cells were maintained in McCoy´s 5A modified medium with 10% FBS and 1% penicillin-streptomycin. HeLa, Panc-1, MCF-7, MelJuSo, TGR-1, HO15.19 and HO-MYC3 cells were cultured in DMEM medium supplemented with 10% FBS and 1% penicillin-streptomycin. All cells were maintained at 37°C in a humidified incubator with 5% CO_2_.

### SDS PAGE and western blotting

Cells were lysed using RIPA buffer supplemented with protease inhibitors (Sigma). Proteins were fractionated using NuPAGE gels (Novex, Life Sciences) and transferred to nitrocellulose membranes. Membranes were blocked in 5% milk and incubated overnight with primary antibodies. Antibodies used were as follows, β-actin (Sigma-Aldrich), MYC, 4EBP1, phospho-4EBP1, CRD-BP/IMP1 (Cell Signaling). Proteins were visualized using ECL chemiluminescence.

### Generation of spheroids

Spheroids were prepared essentially as previously described [55]. In brief, a cell suspension containing 10,000 cells (200 μl) was added to each well of poly-HEMA-coated 96-well plates. Wells were overfilled by adding 170 μl media to acquire a convex surface curvature and plates were inverted to allow cells to sediment to the liquid–air interface. Plates were returned to normal after 24 h incubation, excess media were removed by aspiration and incubated for 4 days prior to drug exposure.

### Measurements of oxygen consumption rate (OCR)

Mitochondrial function was analyzed by measuring oxygen consumption rates (OCR) using a Seahorse XF analyzer according to manufacturer's instructions (Seahorse Bioscience, North Billerica, MA, USA). In brief 60,000 cells/per well were plated in 100 μl culture medium in XF24-well cell plates. Prior to measurement of OCR, medium was replaced with 500 μl Seahorse assay media (1 mM pyruvate, 25 mM glucose and 2 mM glutamine). Oligomycin, FCCP, rotenone and antimycin A were injected in sequence. All experiments were performed at least 3 times.

### RNA extraction, reverse transcription, and real-time PCR

Total RNA was isolated from cell pellets using the RNeasy Isolation kit (Qiagen). Total cDNA was synthesized using reverse transcription kit (Invitrogen) according to manufacturer's instructions. Determination of gene expression levels was performed using TaqMan^®^ Fast Advanced Master Mix (Applied Biosystems) and probes for each subunit of tubulin (Hs00742828_s1) and *MYC* (Hs00153408_m1) (Life Technologies).

### MicroRNA array analysis

For microarray analysis, HeLa cells were treated with VLX600 (6.5 μM) or FCCP (10 μM) for 12 h. Cells were then lysed and total RNA was isolated using RNeasy Plus Mini Kit (Qiagen, Hilden, Germany) according to manufacturer instructions. 500 nM total RNA was biotin labeled using the Flashtag^™^ Biotin HSR RNA Labeling Kit (Affymetrix, Santa Clara, CA). Successful biotin labeling was confirmed using the included ELISA QC assay. Biotin labeled RNA was hybridized to GeneChip^®^ miRNA 4.0 chips for 16 h in a GeneChip^®^ Hybridization Oven 645, washed and stained using the GeneChip^®^ Fluidics Station 450 using the FS450_0003 protocol and scanned with a GeneChip^®^ Scanner 3000 7G (Affymetrix, Santa Clara, CA). Expression data was normalized using RMA-DABG analysis in the Affymetrix^®^ Expression Console Software build 1.4.1.46. Changes in expression were visualized using Affymetrix^®^ Transcriptome Analysis Console (TAC) Software version 3.1.0.5 (both Affymetrix, Santa Clara, CA).

### Let-7 family/miR34a-5p inhibitor transfection

HeLa cells were plated one day before transfection, and transfected with either 15 nM miRCURY LNATM miRNA inhibitor control, 15 nM hsa-let-7 miRCURY LNA^™^ miRNA Power family inhibitor or 15 nM hsamiR34a5p miRCURY LNATM Power miRNA inhibitor (EXIQON), using Lipofectamine Messengermax (Thermo Fisher Scientific), for 24 hours prior to analysis.

### Cell growth assay

HeLa cells were plated in 6-well plates 16 hour prior to transfection at a concentration of 100,000 cells per well. Cells were transfected with control LNA, *let-7* family power inhibitor LNA or *miR-34a-5p* LNA (Exiqon) at 15 nM per well in triplicates. Cells were reseeded at a concentration of 50,000 cells per well 24 hour post transfection and treated with 6.5 μM VLX600 or DMSO. Cells were allowed to expand for two and four days before assayed for absolute cell number counts using a Beckman Coulter Z2 Cell and Particle counter. Cells in a diameter range from 12 to 30 μm were counted.

### Generation and confirmation of Rho0 cells

HeLa cells were cultured in DMEM medium supplemented with 100 ng/mL EtBr and 50 μg/mL uridine for one week. DNA was isolated using PureLink^®^ Genomic DNA Mini Kit (Thermo Fisher Scientific) and mtDNA and nDNA were amplified by Human Mitochondrial DNA (mtDNA) Monitoring Primer Set (Takara). Expression levels of genes of interest were measured by 7500/7500 Fast Real-Time PCR System (Applied Biosystems) and final copy numbers mitochondria DNA and nuclear DNA were calculated. *Rho0* cells were also confirmed by western blotting using anti-COX2 (Abcam).

## SUPPLEMENTARY MATERIALS FIGURES AND TABLES


