# Supplementary material for: MYC is downregulated by a mitochondrial checkpoint mechanism
Source: Oncotarget. 2017 Oct 6;8(52):90225–37. doi: 10.18632/oncotarget.21653 (PMC5685744; doi:10.18632/oncotarget.21653)
Supplement: Supplementary file 1 [file oncotarget-08-90225-s001.pdf]

MYC is downregulated by a mitochondrial checkpoint mechanism

SUPPLEMENTARY MATERIALS

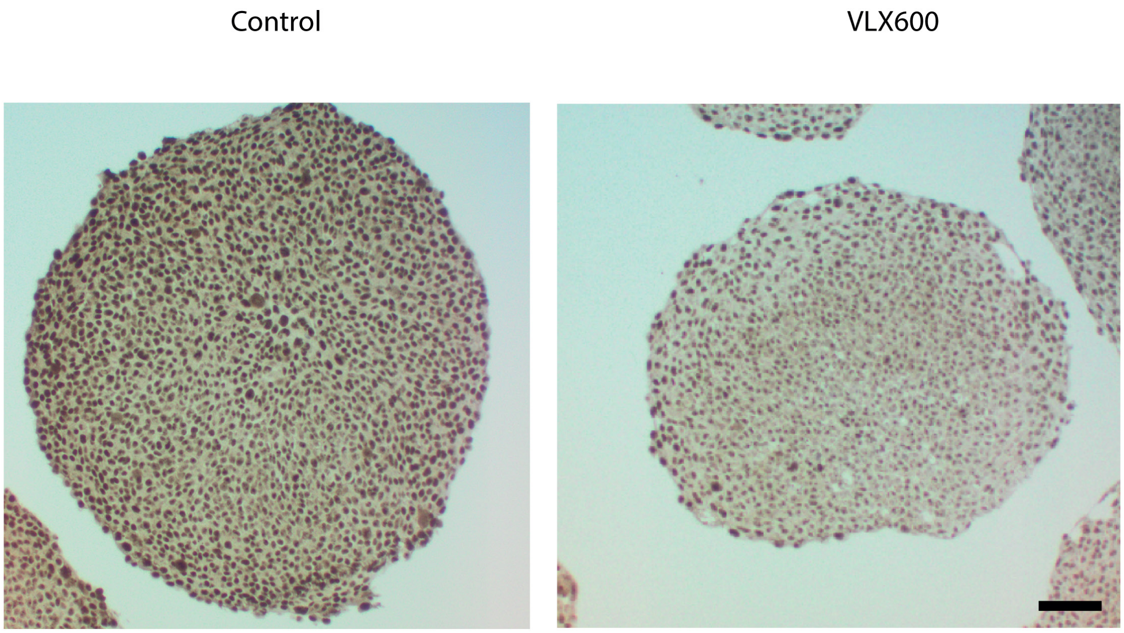

Supplementary Figure 1: VLX600 decreases MYC protein expression in HCT116 colon cancer multicellular spheroids. Spheroids were treated with 6.5 µM VLX600 for 24 hours followed by immunohistochemistry staining for MYC expression. Bar scale: 100 µm.

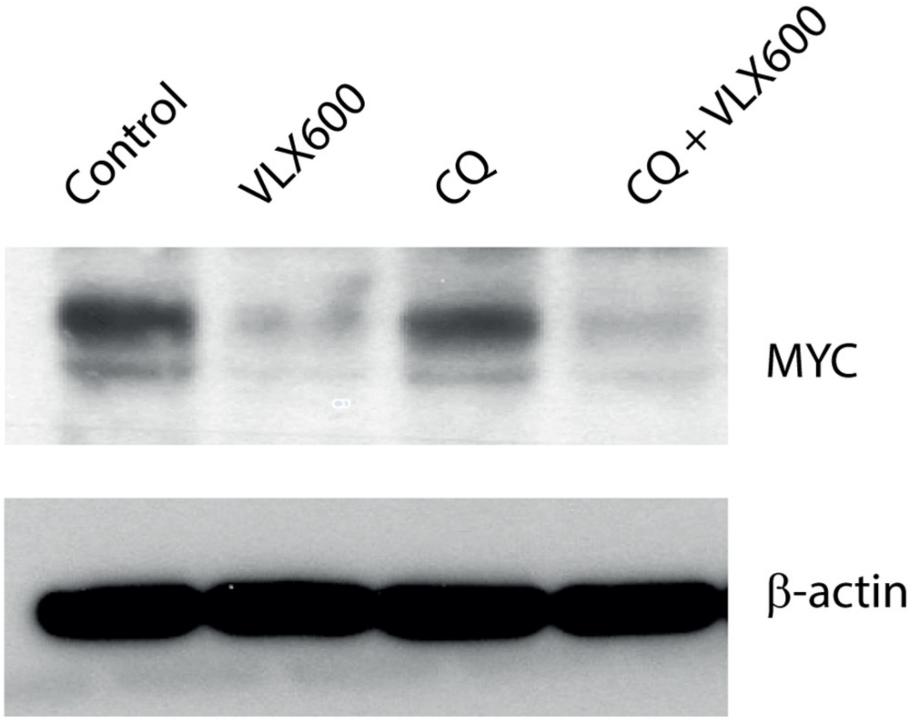

Supplementary Figure 2: VLX600 decreases MYC protein expression independent of autophagy. HCT116 cells were treated with VLX600, CQ (autophagy inhibitor), or indicated drug combinations for 24 h. Note that inhibition of autophagy does not restore MYC levels.

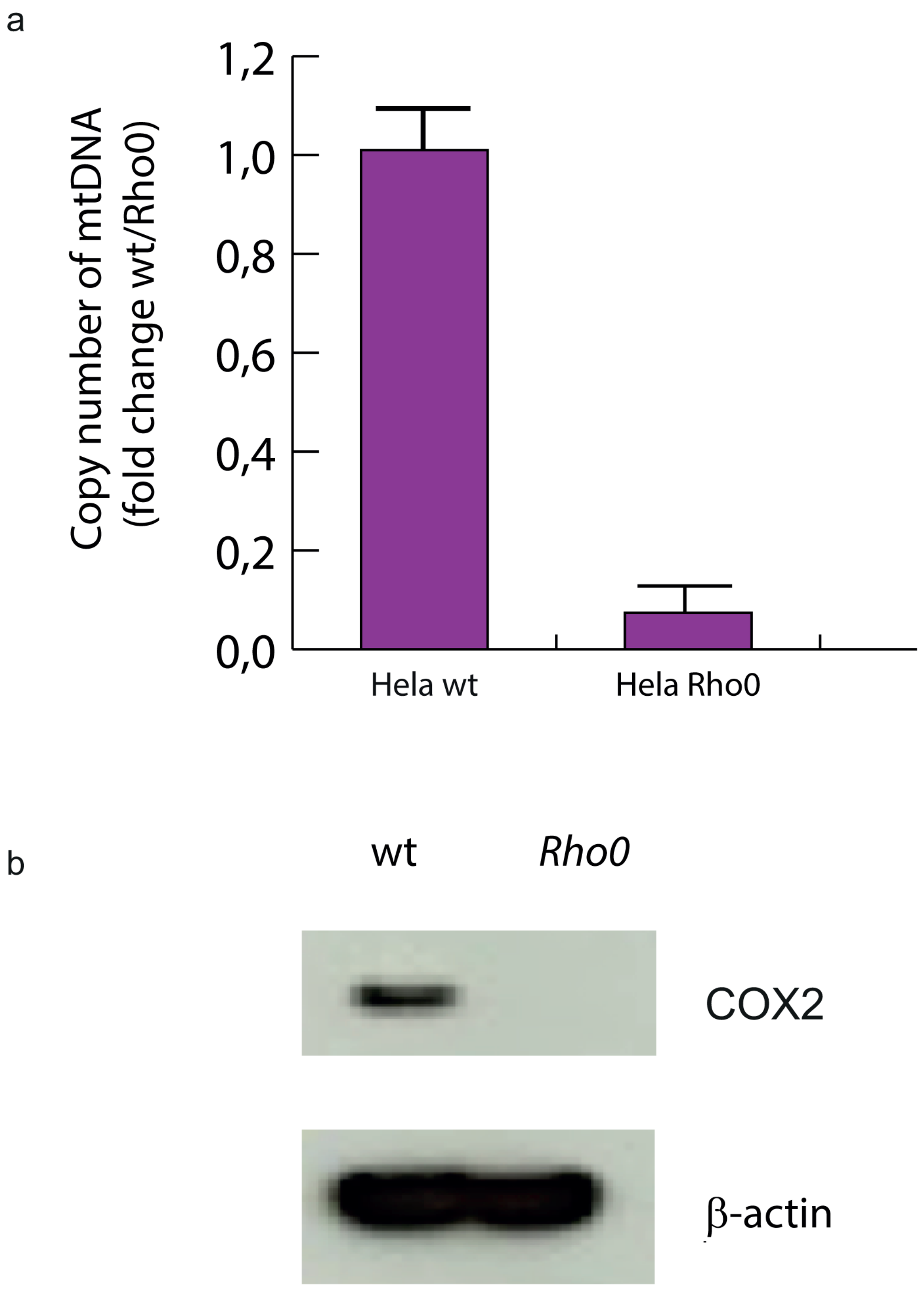

Supplementary Figure 3: Generation of mitochondria depleted Rho0 HeLa cells. HeLa cells were treated with ethidium bromide to generate Rho0 cells. (a) Mitochondrial DNA copy number was determined by PCR as described in Methods and normalized to control cells; (b) analysis of COX2 expression by western blotting (COX2; MTCO2, encoded by the mitochondrial genome).
